# Optimization of cardiopulmonary bypass prime fluid to preserve microcirculatory perfusion during on-pump coronary artery bypass graft surgery: PRIME study protocol for a double-blind randomized trial

**DOI:** 10.1186/s13063-024-08053-5

**Published:** 2024-03-26

**Authors:** Anne M. Beukers, Carolien S. E. Bulte, Ruben J. Bosch, Susanne Eberl, Charissa E. van den Brom, Stephan A. Loer, Alexander B. A. Vonk

**Affiliations:** 1 Department of Anaesthesiology, Amsterdam UMC, VU University Amsterdam, Boelelaan 1117, Amsterdam, The Netherlands; 2 Amsterdam Cardiovascular Sciences, Amsterdam UMC, Amsterdam, The Netherlands; 3 Department of Cardiothoracic Surgery, Amsterdam UMC, University of Amsterdam, Meibergdreef 9, Amsterdam, The Netherlands; 4 Department of Anesthesiology, Amsterdam UMC, University of Amsterdam, Meibergdreef 9, Amsterdam, The Netherlands; 5 Laboratory for Experimental Intensive Care and Anesthesiology (LEICA), Amsterdam, UMC, University of Amsterdam, Amsterdam, The Netherlands; 6 Department of Intensive Care Medicine, Amsterdam UMC, University of Amsterdam, Amsterdam, The Netherlands

**Keywords:** Cardiopulmonary bypass, Priming, Microcirculation, Colloid, Crystalloid, Albumin, Glycocalyx, Colloid oncotic pressure, Gelofusine

## Abstract

**Background:**

Acute microcirculatory perfusion disturbances and organ edema are important factors leading to organ dysfunction during cardiac surgery with cardiopulmonary bypass (CPB). Priming of the CPB system with crystalloid or colloid fluids, which inevitably leads to hemodilution, could contribute to this effect. However, there is yet no optimal evidence-based strategy for this type of priming. Hence, we will investigate different priming strategies to reduce hemodilution and preserve microcirculatory perfusion.

**Methods:**

The PRIME study is a single-center double-blind randomized trial. Patients undergoing elective coronary artery bypass graft surgery with CPB will be randomized into three groups of prime fluid strategy: (1) gelofusine with crystalloid, (2) albumin with crystalloid, or (3) crystalloid and retrograde autologous priming. We aim to include 30 patients, 10 patients in each arm. The primary outcome is the change in microcirculatory perfusion. Secondary outcomes include colloid oncotic pressure; albumin; hematocrit; electrolytes; fluid balance and requirements; transfusion rates; and endothelial-, glycocalyx-, inflammatory- and renal injury markers. Sublingual microcirculatory perfusion will be measured using non-invasive sidestream dark field video microscopy. Microcirculatory and blood measurements will be performed at five consecutive time points during surgery up to 24 h after admission to the intensive care unit.

**Discussion:**

PRIME is the first study to assess the effect of different prime fluid strategies on microcirculatory perfusion in cardiac surgery with CPB. If the results suggest that a specific crystalloid or colloid prime fluid strategy better preserves microcirculatory perfusion during on-pump cardiac surgery, the current study may help to find the optimal pump priming in cardiac surgery.

**Trial registration:**

ClinicalTrials.gov NCT05647057. Registered on 04/25/2023. ClinicalTrials.gov PRS: Record Summary NCT05647057, all items can be found in the protocol.

## Administrative information

**Table:** 

Title {1}	Optimization of prime fluid strategy to preserve microcirculatory perfusion during on-pump coronary artery bypass graft surgery: PRIME study protocol for a double-blind randomized controlled trial
Trial registration {2a and 2b}	ClinicalTrials.gov NCT05647057. Registered on 04/25/2023
Protocol version {3}	Protocol version 4.0, date 04/06/2023
Funding {4}	The PRIME study received no external funding. Funding was obtained by the participating departments from the sponsoring institution
Author details {5a}	^1^Department of Anaesthesiology, Amsterdam UMC, VU University Amsterdam, Boelelaan 1117, Amsterdam, The Netherlands; ^2^Amsterdam Cardiovascular Sciences, Amsterdam UMC, Amsterdam, The Netherlands ^3^Department of Cardiothoracic Surgery, Amsterdam UMC, University of Amsterdam, Meibergdreef 9, Amsterdam, The Netherlands; ^4^Department of Anesthesiology, Amsterdam UMC, University of Amsterdam, Meibergdreef 9, Amsterdam, The Netherlands; ^5^Laboratory for Experimental Intensive Care and Anesthesiology (LEICA), Amsterdam UMC, University of Amsterdam, Amsterdam, the Netherlands; ^6^Department of Intensive Care Medicine, Amsterdam UMC, University of Amsterdam, Amsterdam, the Netherlands
Name and contact information for the trial sponsor {5b}	Department of Anesthesiology, Amsterdam UMC, VU University Amsterdam, De Boelelaan 1117, 1081 HV Amsterdam, The Netherlands
Role of sponsor {5c}	The sponsor had led the study design; collection, management, analysis and interpretation of the data; writing of the report, and the decision to submit the report for publication

## Introduction

### Background and rationale {6a}

Disturbed microcirculatory perfusion is a well-known phenomenon in cardiac surgery with cardiopulmonary bypass (CPB) [1, 2]. Microcirculatory perfusion remains disturbed up to 72 h after surgery and is associated with organ dysfunction [2–4]. This dysfunction is seen in up to 42% of patients and can result in a sixfold increase in mortality rate [5–8].

The underlying cause of microcirculatory perfusion disturbances is an increase in endothelial permeability and vascular leakage, both consequences of systemic inflammation, endothelial activation, hemodilution, and hemolysis [3, 9, 10]. Both, increased permeability and vascular leakage result in an accumulation of interstitial fluid and edema, which can compromise microcirculatory perfusion and tissue oxygenation [2, 11].

With CPB, microcirculatory perfusion directly reduces by 20% after the onset, irrespective of systemic hemodynamics [12], whereby hemodilution is an important factor attributing to this disturbed perfusion pattern [13]. Therefore, the choice of an optimal prime fluid strategy to minimize hemodilution and interstitial fluid accumulation plays an important role. Different prime fluid strategies have been used in order to achieve these goals [14]. These strategies focus on preserving colloid oncotic pressure (COP), a key determinant of interstitial fluid accumulation [15–19].

However, there is as yet no evidence-based strategy for the type of priming. Hence, we will investigate whether different priming strategies reduce hemodilution and hemolysis and preserve microcirculatory blood flow.

### Objectives {7}

The objective is to investigate the effect of three different prime fluid strategies on microcirculatory perfusion in patients undergoing coronary artery bypass graft surgery (CABG) with CPB. The primary outcome is the change in microcirculatory perfusion. Secondary outcomes include colloid oncotic pressure; albumin; hematocrit; electrolytes; fluid balance and requirements; transfusion rates; endothelial-, glycocalyx-, inflammatory- and renal injury markers.

### Trial design {8}

The trial design is a prospective, single-center, double-blind randomized trial (PRIME study). The PRIME study is a three-armed superiority interventional study comparing different prime fluid strategies on microcirculatory perfusion. The participant and investigator are blinded for the intervention. Caregivers will not be blinded, because close communication between all players is of the utmost importance for the safety of the patients, including during retrograde autologous priming.

## Methods

### Study setting {9}

The PRIME study (NCT05647057, ClinicalTrials.gov) will be conducted at the Amsterdam University Medical Centre, location AMC, an academic hospital in The Netherlands. Adult patients undergoing elective on-pump coronary artery bypass surgery will be identified. Participants will be randomized to receive either a combined gelofusine and crystalloid, albumin and crystalloid, or solely crystalloid-based prime fluid strategy, the latter with retrograde autologous priming. This trial protocol uses the Standard Protocol Items: Recommendations for Interventional Trials (SPIRIT) reporting guidance [20].

### Eligibility criteria {10}

Independent investigators will screen all patients scheduled for on-pump coronary artery bypass graft surgery to identify those eligible for participation in this trial. Patients are eligible if they meet the following inclusion criteria:

-  ≥ 18 years of age
- Elective coronary artery bypass graft surgery with CPB
- Informed consent

Exclusion criteria are:

- Emergency operation
- Elective thoracic aortic surgery
- Elective valve surgery
- Combined procedure coronary artery bypass graft surgery and valve surgery
- Known allergy for human albumin or gelofusine
- The use of crystalloid cardioplegia

### Who will take informed consent {26a}

Written informed consent will be obtained preoperatively, after a reasonable reflection period, by a trained and ethical certified investigator at the ward.

### Additional consent provisions for collection and use of participant data and biological specimens {26b}

Participants will be asked for consent, specifically regarding the storage of data, bodily materials, and the use of biological material for future analyses.

## Intervention

### Explanation for the choice of comparators {6b}

Traditionally, priming fluid for CPB comprises crystalloids, colloids, or a combination of both. Crystalloids, like Ringers and lactated Ringers, lead to an increase in interstitial fluid accumulation [21]. Colloids, for example, gelofusine or hydroxyethyl starch, increase COP more than crystalloids, and subsequently reduce fluid requirements during CPB [22]. Previous studies have demonstrated that human albumin and colloids as hydroxyethyl starch prevented an increase in extravascular lung water index compared to crystalloids [15, 17]. However, hydroxyethyl starch was removed from the European market in 2013 by the European Medicines Agency due to safety concerns regarding the increased risk of renal injury associated with its use. The use of albumin in prime fluid strategies might prove its advantage due to its possible protective effect on the endothelial glycocalyx, its ability to preserve COP during CPB, and therefore, its potential to reduce interstitial fluid accumulation [23].

Another frequently used technique in CPB priming is retrograde autologous priming (RAP). Using RAP, the CPB pump is filled with the patient’s own blood in a retrograde manner. RAP reduces the impact of hemodilution by lowering priming volume and compensating for the decline in COP during on-pump cardiac surgery. This leads to a reduction in transfusion rates, and therefore, the use of RAP is recommended in the guidelines on CPB in adult cardiac surgery [16, 24, 25].

Strikingly, the effect of different prime fluid strategies on microcirculatory perfusion during on-pump cardiac surgery has, to the best of our knowledge, never been investigated. Therefore, this study investigates the effects of colloid-based or crystalloid-based prime fluids with RAP on microcirculatory perfusion in on-pump cardiac surgery.

### Intervention description {11a}

Patients will be randomized into three groups with different priming fluids:

- Group A, 750 mL modified fluid gelatin (Braun Melsungen AG, Germany), 650 mL Ringer’s solution (Baxter BV, Utrecht, Netherlands), and 100 mL mannitol (15%, Baxter BV, Utrecht, Netherlands)
- Group B, 200 mL human albumin (20%, Sanquin, Amsterdam, Netherlands), 1200 mL Ringer’s solution (Baxter BV, Utrecht, Netherlands), and 100 mL mannitol (15%, Baxter BV, Utrecht, Netherlands)
- Group C, 1400 mL Ringer’s solution (Baxter BV, Utrecht, Netherlands) and 100 mL mannitol (15%, Baxter BV, Utrecht, Netherlands) with retrograde autologous priming.

RAP is applied in group C using clinical parameters such as central venous pressure, mean arterial pressure, and left ventricular filling assessed with the use of transesophageal echocardiography. RAP is applied to a maximum volume of 475 mL (body surface area (BSA) > 1.7 m^2^) and 375 mL (BSA < 1.7 m^2^) provided that systolic blood pressure will remain > 90 mmHg. Phenylephrine can be administered up to 200 mcg to keep the system hemodynamics stable during RAP. Once the desired amount of prime is displaced, the transfusion bag is clamped and CPB is started. If additional fluids are needed during CPB to maintain optimal perfusion, the displaced prime is used prior to the vasoplegia protocol.

### Criteria for discontinuing or modifying allocated interventions {11b}

Patients can withdraw consent at any time for any reason. If patients are withdrawn prior to surgery, such as for transportation to another hospital or due to a lack of intensive care capacity, they will be replaced. The investigator can decide to withdraw a subject from the study for urgent medical reasons, such as an anaphylactic reaction. In case of an anaphylactic reaction on modified fluid gelatin or human albumin, patients will receive 100 mg hydrocortisone and optional 100 mcg adrenalin based on the decision of the anesthesiologist. Patients with an anaphylactic reaction to the components of prime fluids will be withdrawn from the study, and a replacement subject will be enrolled according to the study’s eligibility criteria.

### Strategies to improve adherence to interventions {11c}

To improve adherence to the intervention protocol, the staff members of the cardiothoracic surgery, cardiac anesthesia, and extracorporeal circulation department are informed about the study, through oral presentations at their department by the PhD student (AMB). If a cardiac surgeon wishes to operate with crystalloid cardioplegia instead of blood cardioplegia, the patient will not be included. Patients will not receive reimbursement for study participation, since no additional harm or patient discomfort is expected.

### Relevant concomitant care permitted or prohibited during the trial {11d}

#### Fluid protocol

In case of hemodilution, defined as a hematocrit below 0.24 L L^−1^, packed red blood cells will be transfused. In case of a low cardiac index < 2.2 L min^−1^ m^−2^, patients will receive 500 mL Plasmalyte.

In case of vasoplegia, defined as MAP < 50 mmHg and cardiac index > 2.2 L min^−1^ m^−2^, patients will receive in order (1) noradrenaline 0.02–0.2 mcg kg^−1^ min^−1^, with a phenylephrine bolus 50–500 mcg as bridge to noradrenaline; (2) terlipressin, starting with 1 mcg kg^−1^ h^−1^, raising dosage with 0.5 mcg kg^−1^ h^−1^; and (3) methylene blue 1–2 mg kg^−1^ in 100 ml sodium chloride 0.9%. Before and after CPB, patients will receive Plasmalyte based on the decision of the anesthesiologist. Besides the intervention groups, no additional colloid fluids will be given in the perioperative process.

#### Anesthesia and CPB protocol

After induction of anesthesia using intravenous sufentanil (0.25–0.5 μg kg^−1^), propofol (0.5–2 mg kg-^1^), lidocaine (1–1.5 mg kg^−1^), midazolam (0.05–0.1 mg kg^−1^), and ketamine (0.25–0.5 mg kg^−1^) combined with rocuronium bromide (0.6–1.2 mg kg^−1^) and maintained by continuous propofol infusion (4–6 mg kg^−1^ h^−1^) and continuous sufentanil infusion (0.5–1 mcg kg^−1^ h^−1^). After tracheal intubation, the lungs are ventilated with a tidal volume of 6–8 ml kg^−1^ resulting in an end-tidal CO_2_ concentration between 4 and 5%. An O_2_-air mixture with an FiO_2_ of 0.4–0.5 and a positive end-expiratory pressure of 5 cm H_2_O will be used. After induction of anesthesia, all patients receive cefazolin (2 g), dexamethasone (0.5 mg kg^−1^ mg), and clemastine (2 mg). In case of blood loss > 2 L or duration of surgery > 4 h, cefazolin will be repeated. All patients will receive tranexamic acid before initiation of CPB (10 mg kg^−1^) followed by a continuous infusion of 1–2 mg kg^−1^ h^−1^. In case of hyperfibrinolysis measured with rotational thromboelastometry (ROTEM), an additional bolus of 1–2 g will be given. Preoperative volume losses are replaced using Plasmalyte.

In all groups, a C5 or a S5 heart–lung machine (LivaNova Nederland NV, Amsterdam, Netherlands) with a centrifugal pump and a heater-cooler device, HCU 40 (Getinge Nederland, Hilversum, Netherlands), will be used for CPB. The bypass circuit consists of a phosphorylcholine-coated tubing system (LivaNova Nederland BV, Amsterdam, Netherlands) with an oxygenator (Inspire 8F) and arterial line, a soft shell venous reservoir (BMR 1900, Medtronic/LivaNova Nederland NV, Amsterdam, Netherlands), and a HVR cardiotomy reservoir (Medtronic/LivaNova Nederland NV, Amsterdam, Netherlands). The extracorporeal circuit will be primed based on the study groups. A 24-French arterial cannula is placed in the ascending aorta and a (two-stage) venous cannula (36 French/46 French) is placed in the right atrium after administration of heparin (300 IE/kg). Venting of the aorta will be achieved by an aortic root cannula. When the activated clotting time exceeds 400 s (Hemochron Signature Elite, Edison, USA), it is considered safe to initiate bypass. Blood flow during mild hypothermic (34 °C) CPB will be kept between 2.2 and 2.6 L/min/m^2^. Myocardial protection is achieved by autologous warm blood cardioplegia, and weaning from CPB is started when the rectal temperature has reached 36 °C. A cell-saving device (Xtra™ Autotransfusion System, LivaNova Nederland BV, Amsterdam, Netherlands) is used for retransfusion of pericardial shed blood. After weaning from CPB, administration of protamine reverses heparin in a 0.6:1 fashion and is monitored with HepCon and ROTEM analysis.

The CPB is primed by the perfusionist in an aseptic environment at a maximum room temperature of 24°. The CPB, primed with the defined solutions, is used within 24 h after priming the circuit. This shelf life is in accordance with obtained pharmaceutical expert advice, under the previously mentioned circumstances.

### Provisions for post-trial care {30}

The sponsor supplied insurance, even without fault, to cover its liability as the requesting party in the event of harm caused to the patient by participation in the study.

### Outcomes {12}

The primary outcome is the change in perfused vessel density (mm mm^−2^), reflecting microcirculatory diffusion capacity, measured at five consecutive time points: after anesthesia induction, after aortic cross-clamping, after weaning from CPB, after arrival on the intensive care unit (ICU), and 24 h after admission on the ICU.

Secondary outcomes include colloid oncotic pressure (mmHg), albumin (g L^−1^), magnesium (mmol L^−1^), phosphate (mmol L^−1^), glycocalyx shedding-, endothelial-, inflammation-, and renal injury markers: syndecan-1 (ng mL^−1^), heparan sulfate (ng mL^−1^), thrombomodulin (ng mL^−1^), angiopoetin-2 (Ang-2 ng mL^−^), interleukin-6 (IL-6, ng mL^−1^), tumor necrosis factor-alpha (TNF-α ng mL^−1^), neutrophil gelatinase-associated lipocalin (NGAL; ng mL^−1^), hemoglobin (Hb, mmol L^−1^), hematocrit (Ht, L L^−1^), hemolysis index, haptoglobin (g L^−1^), perioperative use of packed red blood cells (PRBCs, mL), fluid balance (mL) and fluid requirements (mL), oxygen delivery index (mL min^−1^ m^−2^), and microcirculatory parameters:

- Total vessel density (mm mm^−2^), density of capillaries reflecting the functional state of the microcirculatory diffusion capacity
- Proportion of perfused vessels (%), binominal determinant of red blood cell velocity: flow or no flow
- Heterogeneity index, reflecting the aspect of heterogeneity of microcirculatory perfusion
- Microvascular flow index, a semi-quantitative assessment of the average red blood cell velocity per quadrant
- De Backer-score, a proxy of total vessel density

All secondary outcomes, except for biomarkers, PRBC use, fluid balance, and fluid requirements, will be measured at five consecutive time points: after induction of anesthesia, after aortic cross-clamping, after weaning from bypass, after arrival on the ICU, and 24 h after arrival on the ICU. Biomarkers will be measured at four consecutive time points: after induction of anesthesia, after weaning from bypass, after arrival in the ICU, and 24 h after arrival in the ICU. Other secondary outcomes will be measured at the end of surgery and during ICU admission. Oxygen delivery index will be measured only during CPB.

Other study parameters include patient demographics (age, sex, body surface area, smoking, diabetes on medication, comorbidities, EuroSCORE II (a preoperative risk evaluations for patients undergoing cardiac surgery based on patient characteristics)), surgical characteristics (type of surgery, CPB time, aortic cross-clamping time, heparin and protamine dosing, activated clotting time), perioperative characteristics (temperature, oxygen saturation, blood pressure, urine production, dose of vasoactive medication (noradrenaline, mcg/kg/min; phenylephrine, mcg; vasopressin, IU/min; methylene blue, mg), serum lactate, serum creatinin levels, estimated glomerular filtration rate, serum hemolysis index and haptoglobin concentration, blood product use, blood loss), postoperative characteristics (the same hemostatic, renal, and volume-related parameters as included intraoperative, duration of mechanical ventilation, length of ICU stay and hospital stay), and postoperative complications (acute kidney injury, respiratory failure, pneumonia, non-preexisting atrial fibrillation, re-do surgery, extra corporeal membrane oxygenation requirement, in-hospital mortality).

### Participant timeline {13}

**Fig. 1:**
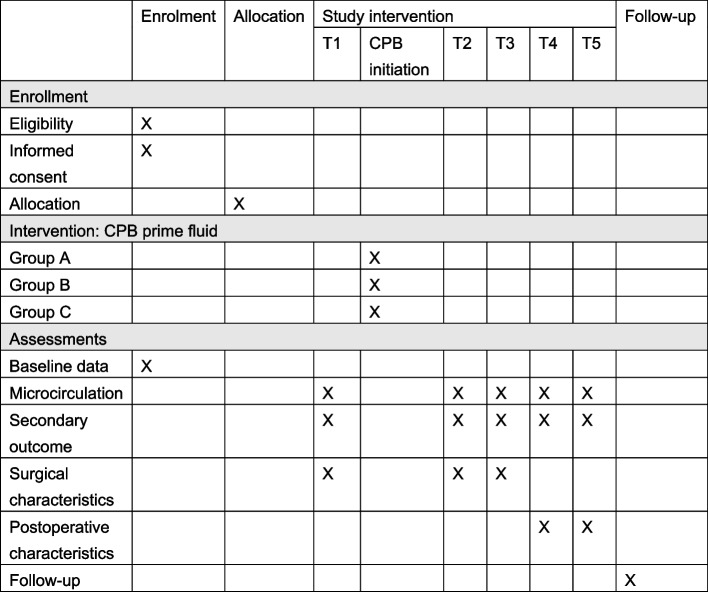
Schedule of enrolment, interventions, and assessment according to SPIRIT. Group A, gelofusine and crystalloid combined; group B, albumin and crystalloid combined; group C, solely crystalloids and retrograde autologous priming. T1, after induction of anesthesia; T2, after aortic cross-clamping; T3, after weaning from bypass; T4, after arrival in ICU; T5, 24 h after arrival in ICU

Patients are screened for eligibility. Thereafter, enrollment and randomization are performed. Figure 1 shows the scheduled enrolment, interventions, and assessment according to SPIRIT.

### Sample size {14}

In previous studies, we found the decrease in perfused vessel density from induction of anesthesia to CPB initiation in our patient population to be 4 and 5 mm mm^−2^, with a standard deviation of 2 mm mm^−2^ [12, 26, 27]. Based on five pairwise comparisons, we set the two-sided significance level used in our power analyses at 0.05. To illustrate a maximal decrease in perfused vessel density of 5 mm mm^−2^ (standard deviation 4) between baseline (T1) and T2–T5 within a group with 80% power, 8 patients are required per group. To account for an anticipated dropout of 20%, a total of 10 patients need to be included. We will include 10 patients in each of the priming groups (30 in total). Secondary study measurements are explorative; hence, no sample size calculation is needed.

### Recruitment {15}

Patients planned for elective coronary artery bypass graft surgery will be assessed for potential eligibility in the preoperative setting. Eligible patients will receive oral and written information regarding the study by a trained and ethical certified investigator. A certificate is obtained with a Good Clinical Practice (GCP) or a Basic Course for ethical regulations for Clinical Researchers (in Dutch: Basis cursus Regelgeving en Organisatie voor Klinische onderzoekers (BROK)) course. The aims of the study, the methods used, and the potential positive and negative effects will be discussed. Patients are given the opportunity to ask questions about this information brochure during and after the preassessment. Questions about study procedures will be answered by the investigator, whether questions regarding routine clinical care in cardiac surgery will be answered by the attending physician. The declaration of consent form will be handed over by an ethical certified investigator. Eligible patients will be included in the study after obtaining written informed consent. An independent physician has been appointed to whom the subjects can also direct their questions.

## Assignment of interventions: allocation

### Sequence generation {16a}

Patients will be randomized into three groups using Castor-based 1:1:1 ratio and the use of randomly varying block sizes of three, six, and nine. Inclusions will continue until the sample size per arm is achieved, in accordance with the randomly varying block sizes of three, six, and nine. The generation of the allocation sequence is generated by Castor (EDC, 2020) and is unavailable to the investigators either involved to enrollment, randomization, or the measurements.

### Concealment mechanism {16b}

A second investigator, uninvolved in enrollment, measurements, analyses, or patient care will conduct the randomization process in Castor (EDC, 2020). The investigator will then communicate the assigned group to the caregivers, including the perfusionist responsible for priming CPB with the intervention assigned to each participant.

### Implementation {16c}

Once participants meet the eligible criteria and written informed consent is obtained, the e-CRF for the screening visit will be filled in fully by the investigator who obtained informed consent. Participants will be sequentially numbered in Castor (EDC, 2020), independent of the intervention group. A second investigator, uninvolved in care, enrollment, measurement, or analyses of outcomes, Castor (EDC, 2020) to randomize the participants and inform the caregivers. The second investigator will ensure the proper allocation of the patient to the intervention group, by informing the involved caregivers including the perfusionist for CPB priming. The investigator is directly involved with the inclusion, measurements, or analyses and will be kept blinded for the intervention group.

## Assignment of interventions: blinding

### Who will be blinded {17a}

The investigator, directly involved with either enrollment and measurements or analyses, and participants will be blinded to the group assignment. Caregivers will not be blinded, because close communication between all caregivers (cardiac anesthetist, cardiac surgeon, and perfusionist) is of the utmost importance for the safety of the patients. Caregivers do not participate in data collection or interpretation.

### Procedure for unblinding if needed {17b}

In case of an anaphylactic reaction, unblinding is facilitated to inform the participant about the anaphylactic reaction. Since caregivers are not blinded, the unblinding will not delay the urgently needed treatment in case of an anaphylactic reaction.

## Data collection and management

### Plans for assessment and collection of outcomes {18a}

Data on the primary outcome will be collected using a non-invasive sidestream dark field (SDF) video microscopy (USB3 with SDF technology, Microvision Medical, Amsterdam, the Netherlands) for visualization of flowing red blood cells and the analysis of microcirculatory perfusion. The SDF camera consists of a hand-held microscope, with the tip surrounded by light-emitting diode illumination. Ensuring the highest absorption of oxy- and de-oxyhemoglobin, at a wavelength of 540 nm, the red blood cells are visible within the capillaries. The tip of the lens is covered by a sterile disposable cap. Imaging of the sublingual microcirculation is carefully performed, with a minimum of 3-s recordings. The technique is performed according to the international guidelines for sublingual microcirculatory measurements [28] and our research group has extensive experience with sublingual microcirculatory measurements during cardiac surgery. Acquired images will be double stored offline for analysis of microcirculatory parameters. Microcirculatory perfusion will be analyzed with a clinically validated software called AVA 4.3. 

Perioperative blood samples will be collected from an existing radial artery catheter in tubes containing dipotassium ethylenediaminetetraacetic acid (K_2_EDTA) (6 mL), heparin-containing tubes (6 mL), and citrate tubes (4 mL). During CPB, blood samples will be collected from a sampling point at the arterial line filter of the extracorporeal circuit. At predefined time points, a total of 80 mL of blood ((6 + 6 + 4 mL) * 5 (measurements) = 80 mL) per patient will be drawn for study purposes. Plasma samples will be stored at – 80 °C until further analysis. COP will be measured with the Osmomat 050 (Gonotec, Berlin, Germany) by means of an oncotic cell. The plasma sample is then injected into the cell (upper) by means of a syringe through a rubber septum. The measuring cell is automatically rinsed with lactated Ringer’s solution. The cell is separated into two halves by a semipermeable membrane, through which only water and electrolyte molecules can permeate. Due to an osmotic pressure difference, solvents from the measuring cell permeate to the upper cell until an equilibrium is reached. An electronic pressure measuring system in the measuring cell transduces the under pressure into an electric signal, which is shown on a display in mmHg. Glycocalyx shedding-, endothelial-, inflammatory-, and renal injury markers are measured using ELISA (SEA565hu and SEB966hu, Cloud-clone Corporation, Hubei, China) in accordance with the manufacturer’s instructions and corrected for corresponding hematocrit levels.

### Plans to promote participant retention and complete follow-up {18b}

To promote participant retention, the participant will be assigned to one investigator who is in contact with the participant during the whole study and who will perform the measurements.

### Data management {19}

Data of individual participating patients will be provided with a subject identification code. The code will be numbered in order of patient entrance into the study. All collected data are protected according to the data protection standards of The Netherlands and the European Union. All data will be entered into an online database Castor (EDC, 2020). In the electronic case report form, we will use a formal and broadly applicable language knowledge representation.

### Confidentiality {27}

Only local hospital investigators, the project leader, and two research coordinators have access to patient data codes, safeguarded by the principal investigator. Access to e-CRF is restricted by user password and role permission access. Logins and passwords are used to protect against unauthorized access to the database. Each user will be assigned to a pre-defined role based on the specific tasks during the study.

### Plans for collection, laboratory evaluation, and storage of biological specimens for genetic or molecular analysis in this trial/future use {33}

Data will be kept for up to 15 years and only authorized organizations have access. Bodily materials will be stored for 5 years. The retention of bodily material is necessary for the purpose of carrying out further investigations during the course of this research. Participants will be asked for consent, specifically regarding the storage of data and bodily materials. Once such use is no longer required, the material will be destroyed. Subject data and bodily materials will not be shared with third parties.

## Statistical methods

### Statistical methods for primary and secondary outcomes {20a}

The data will be locked prior to the analysis and analyzed using R statistical language (https://www.r-project.org). Data will be expressed as percentages (%), as mean ± standard deviation or median [interquartile range] in case of non-normally distributed variables. Normality will be checked by means of normal-probability plots (boxplot, Q-Q plot) and the Kolmogorov Smirnov and Shaprio-Wilk tests. For comparison of normally distributed continuous variables, the independent samples t-test will be used. Continuous non-normally distributed variables will be compared using the Mann–Whitney *U* test. Categorical variables will be compared by chi-square or Fisher’s exact test as appropriate. The primary outcome, the difference in perfused vessel density over time between groups, will be analyzed using linear mixed models. Models will include time as a fixed factor and random effect for the patient. If the time factor is significant, meaning that there is an overall between-group difference, means for different time points will be compared pairwise by means of post hoc test with Bonferroni-adjusted significance levels. Results of the pairwise comparisons of the means for different time points will be rather explorative, since the primary outcome is set on the overall between-group difference. The normality of residuals of the mixed linear models will be checked by means of normal-probability plots. In the case of a non-normally distributed outcome variable, log transformations before mixed linear models will be performed. A *p* value < 0.05 will be considered as statistically significant. Analyses of the secondary outcomes will be performed using linear mixed models, as described above, for other repeatedly measured continuous outcomes. Finally, a linear regression will be used for correlations between two continuous variables.

### Interim analyses {21b}

The study will have a 6-month duration, and interim analyses are not planned.

### Methods for additional analyses (e.g., subgroup analyses) {20b}

A subgroup analysis will be performed to explore acid–base differences between groups and oxygen delivery index levels during CPB.

### Methods in analysis to handle protocol non-adherence and any statistical methods to handle missing data {20c}

In relation to the analyses, an intention-to-treat analysis will be performed. If a participant receives another CPB priming and is then randomized, a per-protocol analysis will be conducted without including these participants. This study group is not powered for a safety analysis; therefore, it will not be performed. To prevent missing data during the trial, participants will receive clear instructions regarding the microcirculatory perfusion measurements, and investigators will be properly trained to perform these measurements. If missing data occurs, the reasons why data are missing will be described in the e-CRF. Missing data will be handled depending on the patterns of missingness, whether they differ across time points or groups. If microcirculatory perfusion measurements are inadequate after baseline, the participant will be excluded from the analyses. If microcirculatory perfusion measurements are inadequate for fewer time points, these will be handled as missing data. The sample size calculation anticipates for a dropout of 20%, for example, based on inadequate microcirculatory perfusion measurements, failure of measurements due to other reasons, or withdrawal of informed consent during follow-up.

### Plans to give access to the full protocol, participant-level data, and statistical code {31c}

The dataset analyzed during the current study and the statistical code are available from the corresponding author upon reasonable request. There are no additives to the protocol available as reported in this manuscript. This article incorporates the full study protocol.

## Oversight and monitoring

### Composition of the coordinating center and trial steering committee {5d}

The composition of the trial steering committee is composed of four members of the sponsor, including the principal investigator and the coordinating investigator. The coordinating investigator receives day-to-day support for the trial from the investigators. The trial steering committee will be monthly informed about how the study is running.

### Composition of the data monitoring committee, its role and reporting structure {21a}

The PRIME study will be conducted in compliance with all relevant Dutch laws and regulations governing the conduct of research involving human subjects, such as the Medical Research Involving Human Subjects Act and the Medical Treatment Contracts Act. Formal auditing is not required. An independent monitor (quality officer) will monitor the study data, with subsequent follow-up assessments as needed.

### Adverse event reporting and harms {22}

All potential adverse events and harms resulting from these interventions will be systematically collected from the electronic patient documents and documented until hospital discharge. Reporting of such events will adhere to the standards set by the Medical Ethical Committee. Serious adverse events will be reported through a web portal (www.toetsingonline.nl) to the Dutch central committee on research involving human subjects (in Dutch: Centrale Commissie Mensgebonden Onderzoek (CCMO)) and the institutional review board (Medical Ethics Committee of Amsterdam UMC). The following serious adverse events must be reported to the study coordinator within 24 h: re-thoracotomy, unplanned re-admission to the ICU, and death (regardless of cause). The remaining adverse events are recorded in an overview list. All adverse events related to the intervention and all serious adverse events will be included in the publication.

### Frequency and plans for auditing trial conduct {23}

An independent monitor is appointed to evaluate the safety parameters. Before the start of the trial, after 25% and 75% of the planned inclusions, the monitor and coordinating investigator will meet in order to assess the safety parameters, to ensure the completeness and accuracy of data. The coordinating investigator will ensure forms on paper as well as electronic are complete.

### Plans for communicating important protocol amendments to relevant parties (e.g., trial participants, ethical committees) {25}

All amendments will be notified to the ethical committee and investigators. Only substantial amendments that affect the safety profile of each participant will be notified to the participants.

### Dissemination plans {31a}

The results of this trial will be submitted to a peer-reviewed medical journal regardless of the study outcome. Authorship will be based on international guidelines.

## Discussion

The PRIME trial is a single-center double-blind randomized trial assessing the effect of three different prime fluid strategies on microcirculatory perfusion during on-pump coronary artery bypass graft surgery. Understanding and preventing microcirculatory perfusion disturbances during on-pump cardiac surgery may reduce organ dysfunction after cardiac surgery. The choice for an optimal CPB priming, which unavoidably leads to hemodilution, may contribute to this outcome. Therefore, this study will examine the effects of three different prime fluid strategies (two colloid-based or crystalloid combined with RAP) on minimizing hemodilution and its impact on microcirculatory perfusion.

Recently, the ALBICS trial, a double-blind large-scale (*n* = 1386) randomized controlled trial on albumin use as prime fluid in cardiac surgery, was published [29]. Albumin 4% was compared with Ringers acetate for CPB priming and fluid resuscitation in the entire perioperative period, and the outcome was the 90-day incidence of major adverse events. No differences between both groups were found. However, exploratory analyses revealed a lower incidence of myocardial injury, and conversely higher bleeding-, re-sternotomy- and infection incidence in the albumin group compared with Ringers acetate. Yet, several aspects of the study could have affected the outcome and reduced the generalizability of the findings. First, the composition and volume of the intervention group (albumin 4% up to 3200 mL excluding 1500 mL prime fluid) differ from clinical practice, as the choice for fluid resuscitation is commonly crystalloids, not solely colloids. Second, albumin impairs hemostasis in a concentration-dependent manner [30]. The high volume of albumin 4% in the entire perioperative period may have affected coagulation. Finally, blood transfusions and coagulation therapies were given based on clinical judgment, rather than transfusion thresholds or coagulation tests (i.e. rotational thromboelastometry). These findings do not provide conclusive evidence about an optimal CPB prime fluid, although earlier studies indicate the positive impact of albumin when included as a prime fluid component [31]. Furthermore, it remains unknown whether a specific crystalloid- or colloid-based prime fluid therapy can effectively preserve microcirculatory perfusion during CPB. However, the ALBICS trial raises important research questions concerning the optimal timing and dosage of crystalloid or colloid fluids in cardiothoracic surgery. While albumin is more expensive than gelofusine, its impact on fluid management, microcirculatory perfusion, and the endothelial glycocalyx in cardiac surgery requires clarification. In addition, the CPB priming volume significantly affects the degree of hemodilution. The use of RAP may mitigate initial priming volume and potentially reduce its impact on microcirculatory perfusion. However, the incorporation of RAP in the crystalloid group may be viewed as a limitation of our study, as initial priming volume could decrease due to RAP. Finally, the use of mannitol remains controversial in literature. Despite international usage being approximately 20% [32] and consistent with our local protocol, we have included mannitol as an additive in all priming groups. In summary, our study investigates the effects of various crystalloid and colloid prime fluids on microcirculatory perfusion in cardiac surgery.

## Trial status

Ethical approval of the primary review committee (Amsterdam UMC) was received on April 19, 2023. The PRIME trial was published in the ClinicalTrials.gov registry on 04/25/2023 (NCT05647057). Approval was obtained for protocol version 4.0, dated 04/06/2023. The first participant was included on July 10, 2023. The anticipated recruitment completion date is expected on May 31, 2024.

## Data Availability

The de-identified data and a study codebook will be made available at the end of the study upon reasonable request to the corresponding author (AMB).

## Abbreviations

CABG: Coronary artery bypass graft surgery
COP: Colloid oncotic pressure
CPB: Cardiopulmonary bypass
ICU: Intensive care unit
RAP: Retrograde autologous priming
ROTEM: Rotational thromboelastometry
